# Diagnosis and treatment of lipodystrophy: a step-by-step approach

**DOI:** 10.1007/s40618-018-0887-z

**Published:** 2018-04-27

**Authors:** D. Araújo-Vilar, F. Santini

**Affiliations:** 1 UETeM-Molecular Pathology Group, Institute of Biomedical Research (CIMUS), School of Medicine, University of Santiago de Compostela, Santiago de Compostela, Spain; 2 Endocrinology Unit, Obesity Center, University Hospital of Pisa, Pisa, Italy

**Keywords:** Diabetes, Dyslipidemia, Insulin resistance, Leptin replacement, Metreleptin

## Abstract

**Aim:**

Lipodystrophy syndromes are rare heterogeneous disorders characterized by deficiency of adipose tissue, usually a decrease in leptin levels and, frequently, severe metabolic abnormalities including diabetes mellitus and dyslipidemia.

**Purpose:**

To describe the clinical presentation of known types of lipodystrophy, and suggest specific steps to recognize, diagnose and treat lipodystrophy in the clinical setting.

**Methods:**

Based on literature and in our own experience, we propose a stepwise approach for diagnosis of the different subtypes of rare lipodystrophy syndromes, describing its more frequent co-morbidities and establishing the therapeutical approach.

**Results:**

Lipodystrophy is classified as genetic or acquired and by the distribution of fat loss, which can be generalized or partial. Genes associated with many congenital forms of lipodystrophy have been identified that may assist in diagnosis. Because of its rarity and heterogeneity, lipodystrophy may frequently be unrecognized or misdiagnosed, which is concerning because it is progressive and its complications are potentially life threatening. A basic diagnostic algorithm is proposed. Effective management of lipodystrophy includes lifestyle changes and aggressive, evidence-based treatment of comorbidities. Leptin replacement therapy (metreleptin) has been found to improve metabolic parameters in many patients with lipodystrophy. Metreleptin is approved in the United States as replacement therapy to treat the complications of leptin deficiency in patients with congenital or acquired generalized lipodystrophy and has been submitted for approval in Europe.

**Conclusions:**

Here, we describe the clinical presentation of known types of lipodystrophy, present an algorithm for differential diagnosis of lipodystrophy, and suggest specific steps to recognize and diagnose lipodystrophy in the clinical setting.

## Introduction

Rare diseases are complex, chronic, often little-known conditions that can have debilitating health consequences and high morbidity and can impair quality of life. They are poorly understood by general practitioners and medical specialists alike, in part because of their low prevalence and frequent phenotypic heterogeneity. Lack of knowledge about rare disorders often leads to long delays in diagnosis and sometimes to misdiagnosis, which can be greatly detrimental to patients.

Lipodystrophy syndromes, a collection of rare heterogeneous disorders characterized by a deficiency of adipose tissue without evidence of nutrition deprivation or a catabolic state, are no exception [1]. These disorders may be associated with a severe form of metabolic syndrome caused by abnormal deposition of fat that cannot be stored in appropriate subcutaneous depots [2]. Loss of adipose tissue frequently results in a decrease in leptin levels [3], which interferes with hunger-satiety signals and often leads to hyperphagia [4]. Surplus calories are stored as fat in liver and muscle tissue, resulting in insulin resistance, hypertriglyceridemia, and hepatic steatosis.

Excluding HIV-related lipodystrophy, the worldwide prevalence of lipodystrophy was recently estimated from searches of large electronic medical record databases to be 3.07 cases per million population [0.23 cases/million of generalized lipodystrophy (GLD) and 2.84 cases/million of partial lipodystrophy (PLD)]. Through literature searches, the prevalence of GLD and PLD was estimated to be 0.96 and 1.67 cases per million population, respectively [5].

The objective of this review was to establish a phased strategy allowing clinicians to attain a reasonably reliable diagnosis of the different lipodystrophy subtypes as well as to outline the therapeutic approach.

## Recognition and diagnosis of lipodystrophy

Recognition of a lipodystrophy disorder is based on clinical history and physical examination that reveal a distinct body composition and metabolic state. Lipodystrophy is classified according to the manner of acquisition (genetic or acquired) and the distribution of adipose deficiency (generalized or partial). Thus, there are four major subtypes: congenital GLD (CGL), acquired GLD (AGL), familial PLD (FPLD), and acquired PLD (APL) (Table 1) [1].

**Table 1:** Classification of lipodystrophies [1, 17, 25–32, 49–58]

1 Congenital
1.1 Generalized (Berardinelli-Seip syndrome)
1.1.1 Type 1 congenital generalized lipodystrophy (*AGPAT2*, recessive, OMIM #608594)
1.1.2 Type 2 congenital generalized lipodystrophy (*BSCL2*, recessive, OMIM #269700)
1.2.3 Type 3 congenital generalized lipodystrophy (*CAV1*, recessive, OMIM #612526)
1.2.4 Type 4 congenital generalized lipodystrophy (*PTRF*, recessive, OMIM #613327)
1.2.5 *PPARG*-associated congenital generalized lipodystrophy (recessive)
1.2 Partial
1.2.1 Type 1 familial partial lipodystrophy (Köbberling syndrome; unknown genes, dominant or polygenic, OMIM **#**608600)
1.2.2 Type 2 familial partial lipodystrophy (Dunnigan disease; *LMNA*, dominant or codominant, OMIM #151660)
1.2.3 Type 3 familial partial lipodystrophy (*PPARG*, dominant, OMIM #604367)
1.2.4 Type 4 familial partial lipodystrophy (*PLIN1*, dominant, OMIM #613877)
1.2.5 Type 5 familial partial lipodystrophy (*CIDEC*, recessive, OMIM #615238)
1.2.6 Type 6 familial partial lipodystrophy (*LIPE*, recessive, OMIM #615980)
1.2.7 *AKT2*-linked lipodystrophy (dominant)
1.2.8 Partial lipodystrophy, congenital cataracts, and neurodegeneration syndrome (*CAV1*, OMIM #606721)
1.3 Systemic
1.3.1 Progeroid syndromes Generalized
1.3.1.1 Hutchinson-Gilford progeria syndrome (*LMNA*, dominant, OMIM #176670) 1.3.1.2 Atypical Werner syndrome and atypical progeroid syndrome (*LMNA*, dominant) 1.3.1.3 SHORT syndrome (*PIK3R1*, dominant, OMOM #269880) 1.3.1.4 MDPL syndrome (generalized or partial; *POLD1*, dominant, OMIM #615381) 1.3.1.5 Keppen-Lubinsky syndrome (*KCNJ6*, dominant, OMIM #614098) 1.3.1.6 Néstor-Guillermo progeria syndrome (*BANF1*, recessive, OMIM **#**614008) 1.3.1.7 Type B mandibuloacral dysplasia (*ZMPSTE24*, recessive, OMIM #608612) 1.3.1.8 Ruijs-Aalfs syndrome (*SPRTN*, recessive, OMIM #616200) 1.3.1.9 Cockayne syndrome (*ERCC6*, *ERCC8*, recessive, OMIM #133540, #216400) Partial 1.3.1.10 Marfan syndrome with neonatal progeroid syndrome–like lipodystrophy (*FBN1*, dominant, OMIM #616914) 1.3.1.11 *CAV1*-associated neonatal onset lipodystrophy syndrome (dominant) 1.3.1.12 Werner syndrome (*WRN*/*RECQL2*, recessive, OMIM #277700) 1.3.1.13 Type A mandibuloacral dysplasia (*LMNA*, recessive, OMIM #248370) 1.3.1.14 *PCYT1A* lipodystrophy (recessive) 1.3.1.15 Bloom syndrome (*RECQL3*, recessive, OMIM #210900) 1.3.1.16 Neonatal progeroid syndrome (possibly associated with *POLR3A*, recessive, OMIM #264090)
1.3.2 Autoinflammatory syndromes, ALDD (generalized or partial), OMIM #256040
1.3.2.1 Nakajo-Nishimura syndrome (*PSMB8*)
1.3.2.2 JMP syndrome (*PSMB8*)
1.3.2.3 CANDLE syndrome (*PSMB8*)
2 Acquired
2.1 Generalized
2.1.1 Acquired generalized lipodystrophy (Lawrence syndrome)
2.2 Partial 2.2.1 HIV-associated lipodystrophy 2.2.2 Acquired partial lipodystrophy (Barraquer-Simons syndrome, OMIM #608709) 2.2.3 Lipodystrophy associated with total body irradiation and hematopoietic stem cell transplant
2.3 Localized
2.3.1 Lipodystrophy caused by drug injections
2.3.2 Lipodystrophy semicircularis
2.3.3 Centrifugal lipodystrophy
2.3.4 Panniculitis-associated lipodystrophy

From: Online Mendelian Inheritance in Man (http://www.omim.org)

*ALDD* autoinflammation, lipodystrophy, and dermatosis syndrome, *CANDLE* chronic atypical neutrophilic dermatosis with lipodystrophy and elevated temperature, *JMP* joint contractures, muscle atrophy, microcytic anemia, and panniculitis-induced lipodystrophy, *MDPL* mandibular hypoplasia, deafness, and progeroid features and lipodystrophy, *OMIM* Online Mendelian Inheritance in Man, *SHORT* short stature, hyperextensibility of joints, hernia, ocular depression, Rieger anomaly, and teething delay

Because of their rarity and heterogeneity, lipodystrophy disorders may frequently be unrecognized or misdiagnosed. Clinicians can differentiate lipodystrophy from other diseases and determine the appropriate subtype using the following steps.

### Step 1: Determination of whether the patient has lipodystrophy

Because of its pervasive features, GLD represents a recognizable phenotype of lipodystrophy. Conversely, the presentation of PLD can be more subtle. PLD may be recognized, in part, by a characteristic pattern of fat loss, described in more detail later in this review [4, 6, 7]. Patients with lipodystrophy may present with the disease in childhood or as adults, and the onset may be sudden or insidious. With few exceptions [8], a major characteristic of lipodystrophy disorders is that fat loss never recovers.

Lipodystrophy should be suspected when a patient presents with a congenital deficiency of subcutaneous adipose tissue (SAT), progressive loss of SAT associated with autoimmune diseases, loss of SAT in limbs concurrent with the accumulation of fat in other body regions, or deficiency of SAT associated with other somatic abnormalities [9]. Additional physical features may include failure to thrive (in children), prominent muscles and veins, acanthosis nigricans, eruptive xanthomas, or cushingoid or acromegaloid appearance [1]. If the patient also has diabetes mellitus requiring high insulin doses, severe hypertriglyceridemia, nonalcoholic steatohepatitis, or polycystic ovarian syndrome (PCOS), lipodystrophy may be further indicated [9].

Diagnosis of lipodystrophy is based on clinical history, physical examination, and assessment of body composition, with laboratory findings useful in some cases. While no firm diagnostic criteria for lipodystrophy have been established based on skinfold measurements or imaging procedures such as dual-energy X-ray absorptiometry and magnetic resonance imaging, all of these evaluations can assist with diagnosis [6, 10–13]. Although serum leptin levels in patients with lipodystrophy tend to be low (in absolute levels or relative to body mass index), no defined serum leptin level threshold can be used to rule out the diagnosis of lipodystrophy [1].

**Fig. 1:**
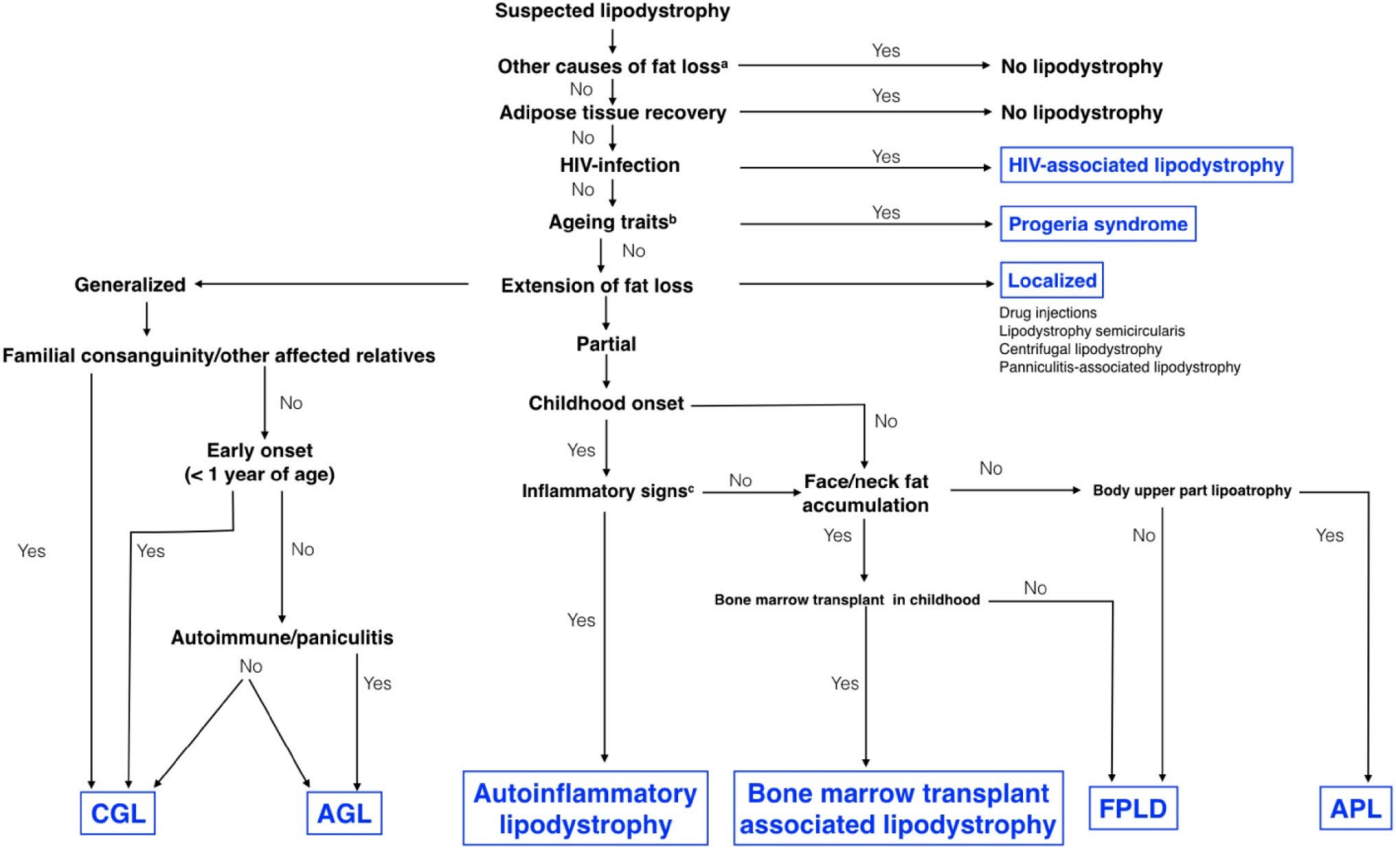
A guide to diagnosing different subtypes of lipodystrophy. Diagnosis of lipodystrophy is largely based on physical examination and clinical history. When abnormal fat loss is observed, other diseases (**a**) causing a negative energy balance should first be excluded (malnutrition, anorexia nervosa, uncontrolled diabetes mellitus, thyrotoxicosis, adrenocortical insufficiency, cancer cachexia, and chronic infections). Recovery of adipose tissue also makes lipodystrophy unlikely. Patients with HIV receiving antiretroviral treatment often develop a syndrome characterized by peripheral lipoatrophy, trunk fat accumulation, and metabolic abnormalities, the pathogenesis of which remains not completely defined. Progeroid syndromes may be associated with lipodystrophy: Aging traits (**b**), such as short stature, alopecia, graying, sclerodermatous skin changes, skin atrophy, osteoporosis, acro-osteolysis, joint contractures, small mandible, dental crowding, low muscle mass, joint stiffness, or mottled pigmentation of the skin, are highly suggestive of early aging syndromes. Of note, fat loss in the face of children may confer an aging appearance that should not be considered a progeroid trait. Once the above-reported conditions are excluded, the extent of fat loss and the distribution of residual adipose tissue must be defined. Localized lipoatrophy may develop due to subcutaneous injection of certain drugs or as a result of regional panniculitis, in the absence of systemic manifestations. Extensive thinning of the subcutaneous adipose tissue should orientate towards a generalized form, whereas incomplete depletion of the adipose pad associated with fat accumulation in the spared regions indicates partial lipodystrophy. Both in generalized and partial lipodystrophies, occurrence of affected relatives or parental consanguinity indicates an inherited subtype of the disease, which should be confirmed by genetic testing. Fat loss in the lower body with fat accumulation in the face and neck is suggestive of FPLD, whereas lipoatrophy of the face that progresses to the shoulder girdle, upper extremities, and trunk is a sign of APL. Coexisting autoimmune diseases, panniculitis, or inflammatory manifestations (**c**: see text) at various sites advocate the diagnosis of acquired lipodystrophy that can be partial at early stages and then evolve as a generalized form. A history of total body irradiation, chemotherapy, and allogeneic bone marrow transplant in the context of leukemia treatment in childhood points to a specific subtype resembling FPLD. *AGL* acquired generalized lipodystrophy, *APL* acquired partial lipodystrophy, *CGL* congenital generalized lipodystrophy, *FPLD* familial partial lipodystrophy

An algorithm for differential diagnosis of lipodystrophy subtypes is shown in Fig. 1. The differential diagnosis includes a range of disparate conditions [9, 14, 15] such as the following:

- Severe weight loss due to anorexia nervosa, starvation, malnutrition, uncontrolled diabetes, hyperthyroidism, adrenal insufficiency, cancer cachexia, or severe chronic infection.
- Donohue syndrome (leprechaunism) and Rabson–Mendenhall syndrome, which are both recessive syndromes associated to variants in the *INSR* gene. Leprechaunism is the most severe disorder characterized by craniofacial abnormalities including elfin face, large, low-set ears, growth retardation, marked lack of adipose tissue, decreased muscle mass, hypertrichosis, pachyderma, acanthosis nigricans, virilization, and severe insulin resistance with paradoxical hypoglycemia. Death often occurs during early infancy. Children with Rabson–Mendenhall syndrome have a longer survival (15–20 years old) and present with coarse face with prognathism, dental crowding, short stature, thin non-lipoatrophic body type, severe acanthosis nigricans, phallic enlargement or clitoromegaly, paradoxical hypoglycemia, hyperinsulinemia, and diabetic ketoacidosis [16].
- Multiple symmetric lipomatosis.
- Cushing syndrome.

### Step 2: Evaluation of the extent of lipodystrophy

Once the diagnosis of lipodystrophy is established, clinicians should investigate whether the disorder is generalized, partial, or localized. In generalized forms, total or near-total loss of subcutaneous fat can be observed over the entire body. In partial forms, fat loss affects large areas, particularly the limbs, but adipose tissue may accumulate in spared regions such as the face and neck in FPLD, while APL is characterized with an opposite distribution of fat. Localized forms of lipodystrophy are limited to small body areas.

After determining whether the lipodystrophy is generalized or partial, a further specification of the particular type of GLD or PLD can be undertaken. Numerous subtypes of GLD and PLD have been described, many associated with known genetic abnormalities (Table 1).

### Step 3.1: Evaluation of GLD

#### CGL

GLD disorders of genetic origin include Berardinelli-Seip syndrome (usually referred to as CGL) and certain disorders of premature aging (progeroid syndromes). A key but nonpathognomonic characteristic to establish the presence of CGL is the age at onset of fat loss, which usually manifests at birth or during the first year of life. However, in some CGL subtypes and progeria syndromes, lipodystrophy arises during childhood.

**Fig. 2:**
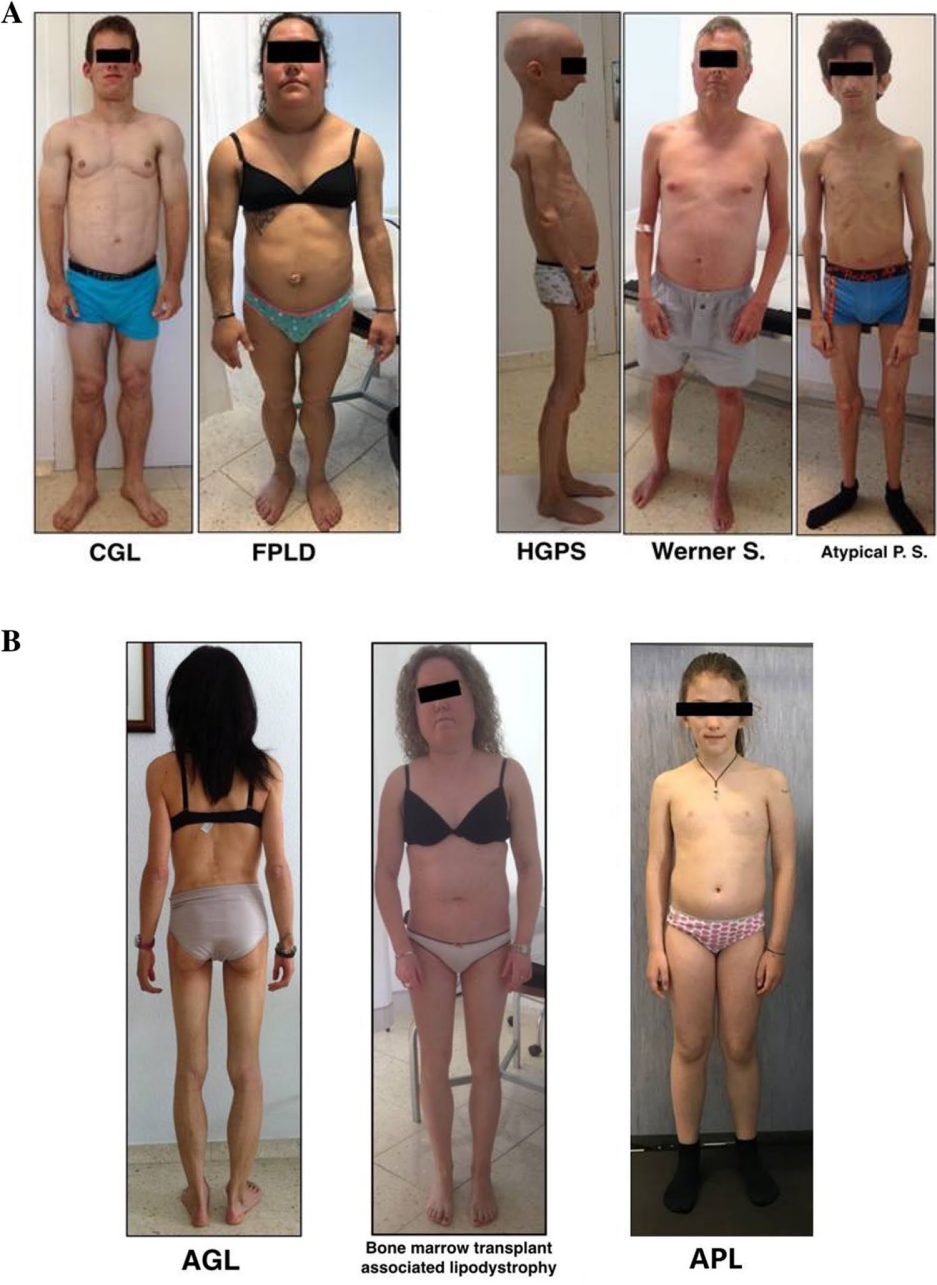
Physical appearance of patients with different lipodystrophy subtypes. **a** Congenital subtypes. Congenital generalized lipodystrophy (CGL): anterior view of a 15-year-old Caucasian man with type 2 CGL due to homozygous c.517dupA (p.Thr173Asn*fs*Ter5) in the *BSCL2* gene; familial partial lipodystrophy (FPLD): anterior view of a 27-year-old Caucasian woman with type 2 FPLD due to heterozygous c.1444C>T (p.Arg482Trp) in the *LMNA* gene; Hutchinson-Gilford progeria syndrome (HGPS): lateral view of a 16-year-old Caucasian woman with HGPS due to de novo heterozygous c.1824C>T (p.[=, Val607_Gln656del]) in the *LMNA* gene; Werner syndrome: anterior view of a 39-year-old Caucasian man with Werner syndrome due to compound heterozygous c.[2886dup]; [1982-5del] (p.[Ile965*]; [Tyr660Ile*fs*7*]) in the *RECQL2* gene; atypical progeria syndrome (PS): anterior view of a 17-year-old Caucasian man with atypical progeroid syndrome due to de novo heterozygous c.29C > T (p.Thr10Ile) in the *LMNA* gene. **b** Acquired subtypes. Acquired generalized lipodystrophy (AGL): posterior view of a 35-year-old Caucasian woman with AGL; bone marrow transplant-associated lipodystrophy: anterior view of a 23-year-old Caucasian woman with partial lipodystrophy associated with bone marrow transplant and total body irradiation during childhood as a treatment for acute leukemia; acquired partial lipodystrophy (APL): anterior view of a 9-year-old Caucasian woman with APL

CGL is a rare autosomal recessive disorder associated with a near total absence of adipose tissue (Fig. 2a) [1, 10]. Patients have marked muscularity, apparent phlebomegaly, and acanthosis nigricans with acrochordons, which frequently extend beyond the axillae and neck and affect the groin, elbow flexion, and abdomen [1, 17]. Patients may exhibit acromegaloid features, beginning in adolescence. Abdominal distension due to hepatomegaly is usually seen from early childhood; hernia or umbilical protrusion is common [1, 9]. In some cases, hypertrichosis is a prominent feature [9]. A voracious appetite is common in early childhood [4].

From the first months of disease, patients with CGL may present with hypertriglyceridemia that, if severe, can lead to acute pancreatitis [9]. Plasma insulin levels are elevated, and nonketotic diabetes, usually appearing in the second decade of life, is often very difficult to control, even with high doses of insulin [9]. Without treatment, the prognosis for patients with CGL is poor because of hepatic cirrhosis, cardiovascular complications of diabetes, pancreatitis, or end-stage renal disease [9].

The mean leptin level in patients with CGL is 1.0 ng/mL and is low regardless of sex and age [18].

In some cases, family history, and almost always phenotypic traits, of patients will help in diagnosis of CGL. Consanguinity should be assessed, because the presence of blood ties in the parents of the propositus would indicate CGL, and the presence of ≥ 1 affected sibling would be almost confirmatory, when associated with phenotypic traits.

Certain clinical features may be associated with the gene responsible for each subtype of Berardinelli-Seip syndrome [1, 9] (Table 1), but genetic testing is required to confirm the type of CGL [1]. Types 1 (*AGPAT2*-associated) and 2 (*BSCL2*-associated) CGL are the most frequent, with type 2 having the most severe metabolic complications and an association with mental retardation [9, 10]. In particular, some variants in *BSCL2* are associated with lethal encephalopathy in early childhood [19]. Patients with CGL and *BSCL2* variants have lower leptin levels and an earlier onset of diabetes than those without these variants [10]. Hypertrophic cardiomyopathy has been reported in types 1 and 2 CGL, as well as accelerated growth and, in women, clitoromegaly and precocious puberty [9, 20]. Conversely, mechanical fat (e.g., palms and soles) is reduced in type 2 CGL, while it is conserved in other types [9, 21].

Additional clinical features such as muscle mounding, muscle weakness, atlantoaxial instability, cardiac arrhythmias, osteopenia, distal metaphyseal deformation with joint stiffness, hypertrophic pyloric stenosis, and esophageal dysmotility can be highly suggestive of type 4 Berardinelli-Seip syndrome [22, 23], associated to variants in the *PTRF* gene.

In early childhood, Berardinelli-Seip syndrome may be confused with Rabson–Mendenhall syndrome and sometimes with Donohue syndrome (leprechaunism). Severe acanthosis nigricans and clitoromegaly can be confounding factors in the diagnosis. However, facial features in those with Rabson–Mendenhall syndrome are distinct and loss of fat, if any, is hardly evident. During adolescence or adulthood, some patients with Berardinelli-Seip syndrome have marked acromegaloid features. Absence of gigantism, generalized loss of subcutaneous fat, apparent hypermusculation, and severe insulin resistance make the differential diagnosis with acromegaly straightforward. If elements of doubt should persist, a normal suppression of serum growth hormone following administration of oral glucose is enough to exclude this disorder [24].

#### Progeroid syndromes

Contrasting with the genetically recessive nature of Berardinelli-Seip syndrome, progeroid syndromes associated with GLD may be dominant (usually de novo) or recessive [25–32] (Table 1). Some features, such as short stature, alopecia, graying, sclerodermatous skin changes, skin atrophy, osteoporosis, acro-osteolysis, joint contractures, small mandible, dental crowding, low muscle mass, joint stiffness, or mottled pigmentation of skin, among others, are highly suggestive of early aging syndromes [33]. For a description of subtypes, see specific references [25–32, 34] (Fig. 2a).

#### AGL

Compared with CGL, AGL (Lawrence syndrome) has a later onset (childhood or adolescence) and is more common in women than men (3:1 ratio) (Fig. 2b) [11]. The loss of adipose tissue occurring in childhood or adolescence, preceded or followed by autoimmune manifestations at various sites, may suggest a diagnosis of AGL [1]. Initially, subcutaneous fat loss may occur at limited locations, but it tends to generalize with progression of the disease over weeks, months, or years. Sometimes, facial fat loss is not initially present, although it usually occurs over time. In some cases, AGL is a phenocopy of Berardinelli-Seip syndrome.

Insulin-resistant diabetes, severe hypertriglyceridemia, hepatic steatosis, and insulin-resistance stigmata are frequent comorbidities of AGL. Hyperinsulinemia and low plasma leptin levels are typically present. Loss of fat in the palms and soles has been reported in approximately one-third and one-half of patients, respectively [11]. There is no family history of lipodystrophy in cases of AGL, but the presence of other autoimmune diseases in relatives may assist in the diagnosis. Activation of the classical complement pathway and low C4 complement levels have been associated with low leptin and adiponectin levels and with destruction of adipocytes and lipodystrophy in patients with AGL [35].

Three subtypes of AGL (panniculitis, autoimmune, and idiopathic) have been proposed [11]. Lipodystrophy onset has been associated with the appearance of panniculitis in ~ 25% of cases and autoimmune disease in another 25% of cases, but no specific causes could be identified in most cases (idiopathic). Patients who developed AGL in association with autoimmune disease tended to be older than those with other AGL subtypes [11]. In particular, the autoimmune disease juvenile dermatomyositis has been associated with AGL [36]. Based on an analysis of a case series, disease associated with panniculitis may progress more slowly than either autoimmune or idiopathic AGL, with a lower prevalence of diabetes and hypertriglyceridemia [11].

### Step 3.2: Evaluation of PLD

The distribution of fat loss, age of onset, certain phenotypic traits, and family history are determining factors in diagnosing the subtypes of PLD, which include congenital and acquired disorders (Table 1).

#### PLD of genetic origin

PLD disorders of genetic origin are described further in the following sections and include FPLD, certain progeroid syndromes, and autoinflammatory syndromes. Many forms of PLD are associated with known genetic abnormalities, and genetic tests are needed to confirm the presence of currently identified mutations in some PLD subtypes [1].

##### FPLD

FPLD encompasses a number of conditions sharing a Cushingoid appearance and a variable association with excess body weight (Fig. 2a). The loss of subcutaneous fat in the limbs and gluteal region, observed around childhood or puberty and associated with accumulation of excess fat in the face, neck, and intra-abdominal region, is suggestive of FPLD [9].

Eight subtypes of FPLD have been reported [17] (Table 1). Type 1 FPLD (Köbberling syndrome) is an early-onset, inherited variety of PLD, although no specific genes are known to be involved, and a dominant or polygenic pathogenesis has been suggested [12, 37, 38]. The diagnosis of type 1 FPLD is challenging because it can be easily confused with android obesity in women associated with metabolic syndrome. Patients with type 1 FPLD are usually obese and have a significant accumulation of abdominal fat, with lipoatrophy most evident in the hips and lower extremities [12]. The disease may represent part of a spectrum encompassing essential central obesity, and specific cutoffs for thickness and distribution of SAT that can be useful for clinical purposes have been proposed [12].

The classic phenotype of FPLD (type 2, Dunnigan disease) is currently attributed to variants in exon 8 of the *LMNA* gene. Fat loss begins around puberty in women and often goes unnoticed in men because of the particular distribution of body fat. Affected men are often diagnosed when an affected female relative is identified. Fat loss is present in the limbs, trunk, hips, and buttocks. Strikingly, these patients have accumulation of fat in the face, neck, axillae, interscapular region, abdominal visceral area, and labia majora [39]. The musculature is well defined and may even include muscular hypertrophy in the calves, as well as apparent phlebomegaly.

Patients with type 2 FPLD, especially women, frequently present with early insulin resistance that may lead to diabetes, hypertriglyceridemia (sometimes severe and causing acute pancreatitis), low high-density lipoprotein cholesterol, hepatic steatosis, and an increased risk of cardiovascular disease; sometimes these patients also present with acanthosis nigricans (although not as severe as in GLD). PCOS is not uncommon, as well as obstetric problems (i.e., gestational diabetes, miscarriage, and stillbirth), muscle aches, and lipomas [39, 40].

Family history (dominant vs. recessive) and certain phenotypic traits and associated disorders may guide molecular diagnosis. For example, in type 2 FPLD there may be valvular heart disease, myocardial hypertrophy, and/or cardiac conduction system disorders. Other laminopathies (Emery-Dreifuss muscular dystrophy, limb-girdle muscular dystrophy, or familial dilated cardiomyopathy) may also be associated with type 2 FPLD or even be present in other family members [41]. Thus, a careful cardiac evaluation is encouraged. Mutations in exons other than exon 8 in *LMNA* can lead to atypical forms of type 2 FPLD, in which lipodystrophy is less evident, or can even be confused with Köbberling syndrome.

In type 3 FPLD, lipoatrophy appears in adulthood, with no fat accumulation in the face or neck. Cardiometabolic complications are usually severe [42]. In type 4 FPLD, lipoatrophy appears in childhood or in adulthood, with possible accumulation of facial fat. These patients also exhibit severe dyslipidemia and insulin-resistant diabetes [43].

Types 5 and 6 FPLD are recessive conditions, and only a few cases have been reported [44–46]. Type 5 FPLD appears in early childhood, while type 6 appears in adulthood. In type 5 FPLD, there is muscular hypertrophy but no accumulation of adipose tissue. Accumulation of fat in the neck, axillae, back, and supraclavicular area; muscular dystrophy (weakness); elevation of creatine kinase; and hypertriglyceridemia have been reported in type 6 FPLD. Because of this particular fat distribution, FPLD must be differentiated from Cushing syndrome. Chronic hypercortisolism causes a characteristic centripetal fat distribution [47], but no particular changes in limb fat have been reported, and muscle wasting is evident [48].

##### Progeroid syndromes

Progeroid syndromes associated with PLD are shown in Table 1 (Fig. 2a) [49–55]. All of these syndromes have particular clinical features of early aging, the detailed description of which is beyond the scope of this review.

##### Autoinflammatory syndromes

Autoinflammatory syndromes causing lipodystrophy include Nakajo-Nishimura syndrome; joint contractures, muscle atrophy, microcytic anemia, and panniculitis-induced lipodystrophy (JMP) syndrome; and chronic atypical neutrophilic dermatosis with lipodystrophy and elevated temperature (CANDLE) syndrome. These disorders begin in childhood, and the lipodystrophy can be generalized or partial, affecting the face and limbs. All are recessive disorders related to mutations in genes encoding for proteins that are essential for the maturation and assembly of the proteasome subunits [56–58]. Normally, type 1 interferon secreted by cells after viral infection or other triggers activates the JAK/STAT pathway that produces reactive oxygen and nitrogen species with defensive function. These oxidant molecules may be detrimental to functional integrity of cell proteins and are usually removed by the proteasome–immunoproteasome system. In autoinflammatory syndromes, cells with mutated proteasome–immunoproteasome will not be able to remove all waste proteins. As a result, an abnormal cellular stress occurs, leading to additional type 1 interferon production and perpetuation of tissue damage [59].

Nakajo-Nishimura syndrome is an inflammatory disease involving lipomuscular atrophy and joint contractures [57]. As indicated by its name, JMP syndrome is characterized by joint contractures, muscular atrophy, microcytic anemia, and panniculitis-induced lipodystrophy [60]. Other characteristics include intermittent fever, hypergammaglobulinemia, increased sedimentation rate, hepatosplenomegaly, and calcification of the basal ganglia. In CANDLE syndrome, patients present with childhood-onset recurrent fevers and violaceous annular plaques on the eyelids and lips, evolving through childhood to a loss of subcutaneous fat in the face and upper limbs. Patients also have hepatosplenomegaly, arthralgias, microcytic anemia, increased sedimentation rate, and calcifications in the basal ganglia [61].

#### APL

APL includes HIV-associated lipodystrophy, Barraquer-Simons syndrome, and bone marrow transplant–associated lipodystrophy. While lipodystrophy in patients with HIV receiving long-term antiretroviral therapy is the most common form of lipodystrophy [62], it is out of the scope of this review.

##### Barraquer-Simons syndrome

Barraquer-Simons syndrome is a very rare disorder of unknown etiology (possibly autoimmune) characterized by a cephalocaudal loss of SAT. It is more common in women than men (4:1 ratio) (Fig. 2b), and fat loss usually begins in late childhood or adolescence. Fat loss initially affects the head, giving children an aged appearance, and progresses to the shoulder girdle, upper extremities, and trunk [1] in a process that can last weeks, months, or years. When affected women gain weight, they accumulate fat in the lower extremities, presenting a unique phenotype of APL. The arms have well-defined musculature and apparent phlebomegaly. Acanthosis nigricans is generally absent [6]. Although the etiology of APL is unknown, the presence of other autoimmune diseases may help confirm the diagnosis, in particular membranoproliferative glomerulonephritis, which can lead to kidney failure [1].

Although reports of metabolic complications have generally been uncommon in Barraquer-Simons syndrome [6], a recent study suggests that these complications have been underestimated [63]. Patients tend to have low serum C3 complement and leptin levels and detectable C3 nephritic factor [6].

##### Bone marrow transplant-associated lipodystrophy

Recently, lipodystrophy following total body irradiation, chemotherapy, and allogeneic bone marrow transplant has been reported as a phenocopy of FPLD [64–66]. This form of APL affects patients who underwent bone marrow transplant in the context of leukemia treatment in childhood (Fig. 2b).

#### Localized lipodystrophy disorders

Localized lipodystrophy disorders involve small amounts of fat loss due to drugs, panniculitis, or unknown etiology [17]. The amount of fat loss is minimal; therefore, these disorders are not associated with metabolic abnormalities.

## Complications of lipodystrophy

Deficient adipose mass in lipodystrophy results in a collection of typical metabolic complications that include insulin resistance and diabetes, hypertriglyceridemia, PCOS, and nonalcoholic fatty liver disease [1]. In patients with lipodystrophy, these complications are sequelae of the defects that led to altered adiposity [1]. Causes of premature mortality include cardiovascular disease, liver disease, kidney failure, pancreatitis, and sepsis [1, 11, 67, 68].

## Treatment approach for lipodystrophy

Lipodystrophy is a progressive and life-threatening disease. Currently, there is no cure for lipodystrophy. Metabolic comorbidities must be treated to manage the short- and long-term complications of the disease.

### Treatment of comorbidities

Diet and exercise are important factors in managing lipodystrophy comorbidities. Practice guidelines recommend that patients adhere to a balanced diet, including ~ 50 to 60% carbohydrates, 20–30% fat, and 20% protein. Because hypoleptinemia (absolute or relative) stimulates appetite and food consumption, energy restriction is usually recommended to reduce ectopic fat storage and improve metabolic abnormalities, although it may be difficult to achieve, especially in childhood and adolescence. Of note, growth assessment may be challenging because of the abnormal body composition and the underlying primary disease that may affect linear growth independent of lipodystrophy. Very low–fat diets should be used in patients with acute pancreatitis. Physical exercise should be advised, if not contraindicated by concurrent diseases, but limitations may intervene because of musculoskeletal pain, fatigue, or psychological distress [1]. Plastic surgery (breast implants, dermal fillers, lipectomy, or liposuction) and psychological support can improve the well-being of some patients.

In addition to lifestyle changes, patients with lipodystrophy should receive medical treatment for specific metabolic comorbidities. Clinicians should follow national or international guidelines in the diagnosis and management of diabetes, dyslipidemia, hypertension, and renal and hepatic disease. As discussed in the next section, leptin replacement therapy may reduce the need for treatment of metabolic comorbidities.

### Leptin replacement therapy

Metreleptin (Myalept^®^, Aegerion Pharmaceuticals, Cambridge, MA, USA) is the only drug indicated specifically for the treatment of lipodystrophy, although many therapies are used to treat comorbid conditions. Metreleptin is a leptin analogue indicated in the United States as an adjunct to diet as replacement therapy to treat the complications of leptin deficiency in patients with CGL or AGL [69]. Metreleptin has been submitted to the European Medicines Agency for approval in the treatment of patients with CGL, AGL, and certain types of PLD.

#### Metreleptin for GLD

Patients receiving metreleptin typically report an immediate decrease in appetite and reduction in food intake [70]. Metreleptin treatment has been associated with an improved metabolic profile in patients with GLD [71]. An open-label cohort study conducted at the National Institutes of Health (NIH) reported reductions in hemoglobin A1c (HbA1c), fasting plasma glucose, and triglycerides within 4 months of initiating treatment, which were maintained for ≥ 3 years in patients with CGL or AGL and at least 1 of 3 metabolic abnormalities (diabetes, insulin resistance, and/or hypertriglyceridemia) [71, 72]. Patients receiving metreleptin were often able to eliminate or greatly reduce the dosage of their antidiabetes medications [73]. The long-term improvements in metabolic parameters continued to accrue even after reduction or discontinuation of antidiabetic therapy [73, 74].

Leptin replacement therapy is also associated with decreased liver volume and serum aminotransferase levels [71, 73, 74]. Paired biopsy studies have shown that nonalcoholic steatohepatitis associated with GLD was ameliorated with metreleptin treatment [75, 76]. During 52 weeks of metreleptin treatment, improved brain connectivity associated with hedonic and homeostatic control of eating behavior, decreased hunger, and increased satiety were observed [77].

#### Metreleptin for PLD

Use of metreleptin in PLD, whether congenital or acquired, is off-label in the United States as of the writing of this review. However, data suggest that the drug may be beneficial in a subset of patients with PLD. Metreleptin treatment was associated with normalization of serum leptin levels within 3 months and decreased triglycerides but not improvement in glycemic control in patients with moderate or severe hypoleptinemia and FPLD (Dunnigan variety) [78]. In the NIH study, metabolic parameters improved over 1 year of treatment with metreleptin in patients with PLD; however, the magnitude of reduction in metabolic parameters was less than that observed in patients with GLD [2].

Some findings suggest that patients with severe PLD may benefit most from leptin replacement therapy. In an analysis from the NIH cohort, metreleptin appeared more effective in patients with higher (vs. lower) baseline metabolic thresholds (baseline HbA1c > 7.0 or > 8.0%, triglycerides > 300 or > 500 mg/dL, and leptin < 4 ng/mL) [2]. In a separate study, a small subset of patients with severe abnormalities at baseline (HbA1c ≥ 8.0% or triglycerides ≥ 500 mg/dL) who were treated with metreleptin for 1 year appeared to derive substantial benefit from treatment compared with the overall treated population [79].

#### Efficacy of metreleptin in children with lipodystrophy

There is some evidence that metreleptin is effective in pediatric patients with GLD or PLD. Improvements in glycemic parameters, triglycerides, liver histology, and markers of liver health were achieved over 1 year of treatment in 53 patients with GLD or PLD and were maintained throughout a mean follow-up of 5 years in the NIH cohort [80]. Metreleptin therapy did not accelerate or trigger puberty, and it was associated with normalization of growth trajectory in this cohort. However, only 8 patients with PLD were included, 7 of which were > 12 years old, which limits generalizing this finding among younger children with PLD.

#### Metreleptin tolerability

The most frequent adverse events associated with metreleptin among patients with GLD (*n* = 48) receiving treatment for a median of 2.7 years were headache (13%), hypoglycemia (13%), and decreased weight (13%) [69]. In the same study, antimetreleptin antibodies were detected in 36 of 43 patients examined, and 2 individuals had neutralizing antibodies, which were associated with infection and loss of metabolic control [69]. Risks of neutralizing antibodies are unclear and should be weighed alongside the benefits of therapy [70].

Lymphoma has been reported in patients with AGL, both in the absence and presence of metreleptin treatment [81]. The increased risk of malignancy in these individuals may be attributable to autoimmune disease; however, a role of metreleptin in tumor growth cannot be ruled out [81].

## Conclusions

Lipodystrophy disorders are a diverse collection of rare congenital or acquired disorders of adipose tissue that range from mild to severe, result in fat loss and metabolic disease, and many are characterized by leptin deficiency. Because lipodystrophy is an ultra-rare disease, it is possible that the practicing clinician will never encounter a patient with GLD or PLD; however, the phenotype and presentation are sufficiently distinct that such patients can be recognized and diagnosed in most instances. Standard of care for lipodystrophy includes treatment with leptin replacement if indicated, aggressive treatment of comorbidities such as diabetes and dyslipidemia, and genetic screening of relatives, if warranted.
